# Supplementary material for: PsAF5 functions as an essential adapter for PsPHB2-mediated mitophagy under ROS stress in Phytophthora sojae
Source: Nat Commun. 2024 Mar 4;15:1967. doi: 10.1038/s41467-024-46290-z (PMC10912746; doi:10.1038/s41467-024-46290-z)
Supplement: Supplementary file 1 — Supplementary Information [file 41467_2024_46290_MOESM1_ESM.pdf]

Supplementary Information

PsAF5 functions as an essential adapter for PsPHB2-mediated mitophagy under ROS stress in *Phytophthora sojae*

Wenhao Li[1], Hongwei Zhu[1], Jinzhu Chen[1], Binglu Ru[1], Qin Peng[1], Jianqiang Miao[1]*, Xili Liu[1,2]*

[1]State Key Laboratory for Crop Stress Resistance and High-Efficiency Production, College of Plant Protection, Northwest A&F University, Yangling 712100, Shaanxi, China

[2]Department of Plant Pathology, College of Plant Protection, China Agricultural University, 2 Yuanmingyuanxi Road, Beijing 100193, China.

*Author for correspondence:

Jianqiang Miao (E-mail: mjq2018@nwafu.edu.cn), Xili Liu (E-mail: seedling@nwafu.edu.cn)

Contents：

Supplementary Figure

Supplementary Table

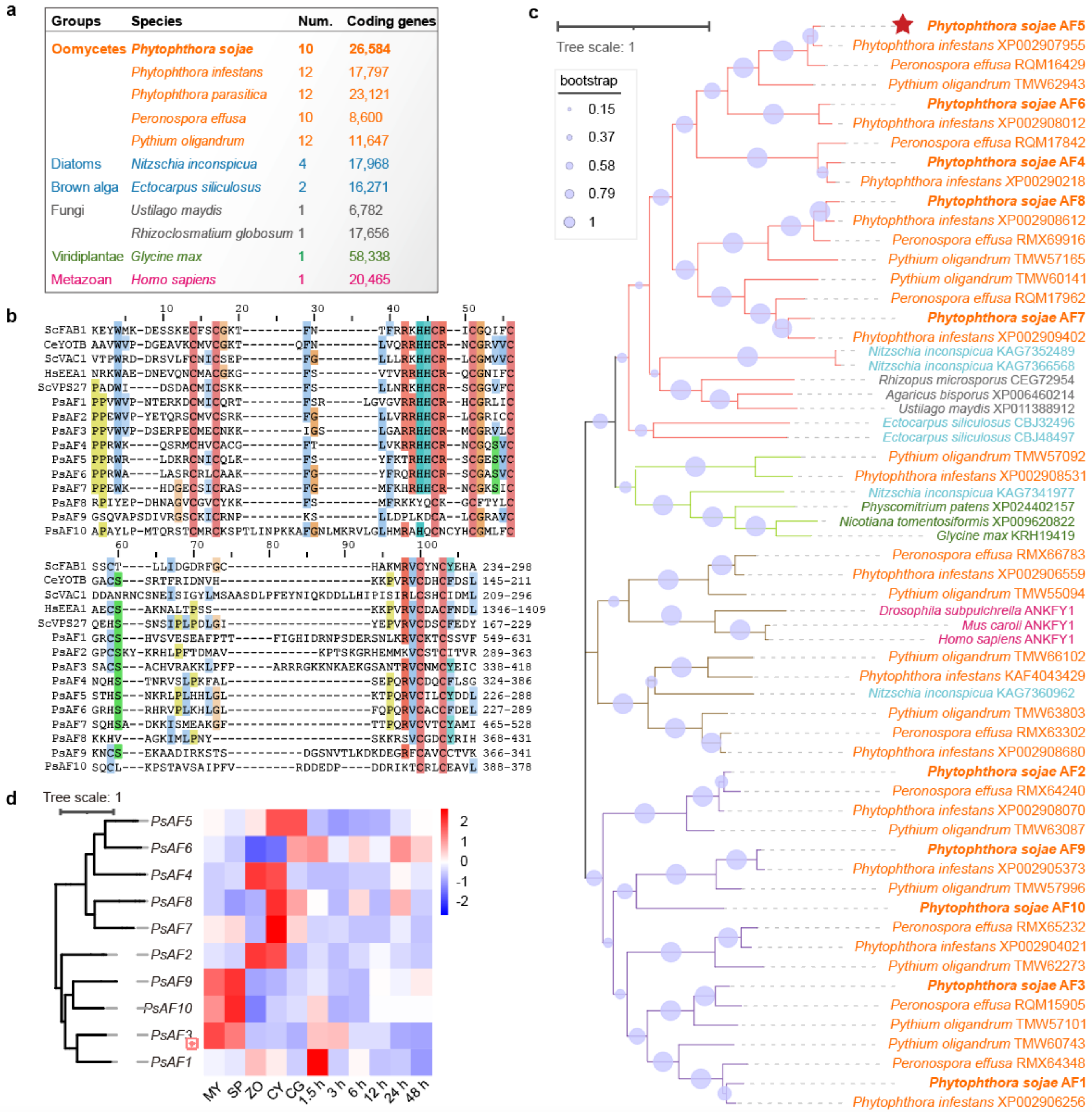

Supplementary Fig 1. Domain conservation and molecular phylogeny of AF proteins, and expression profiles of *PsAF genes*. a Numbers of predicted *AF genes* in various species of oomycetes, fungi, viridiplantae, metazoa, diatoms and brown alga. **b** Multiple sequence alignment of the FYVE domains from *P. sojae* and three other species: *Saccharomyces cerevisiae* (*Sc*), *Caenorhabditis elegans* (*Ce*), and *Homo sapiens* (*Hs*). The amino acid ranges correspond to the positions in each entire protein. **c** Phylogenetic tree showing predicted AF protein families in oomycetes, fungi, viridiplantae, metazoa and diatoms and brown alga. The maximum likelihood method was used. **d** Heat map showing the expression of *PsAF* genes at various stages of growth and infection. MY (mycelia), SP (sporulating hyphae), ZO (zoospore), CY (cysts), and CG (cyst germination)

represent different periods of the developmental stage, whereas 1.5 h, 3 h, 6 h, 12 h, 24 h, and 48 h represent different periods of the infection stage.

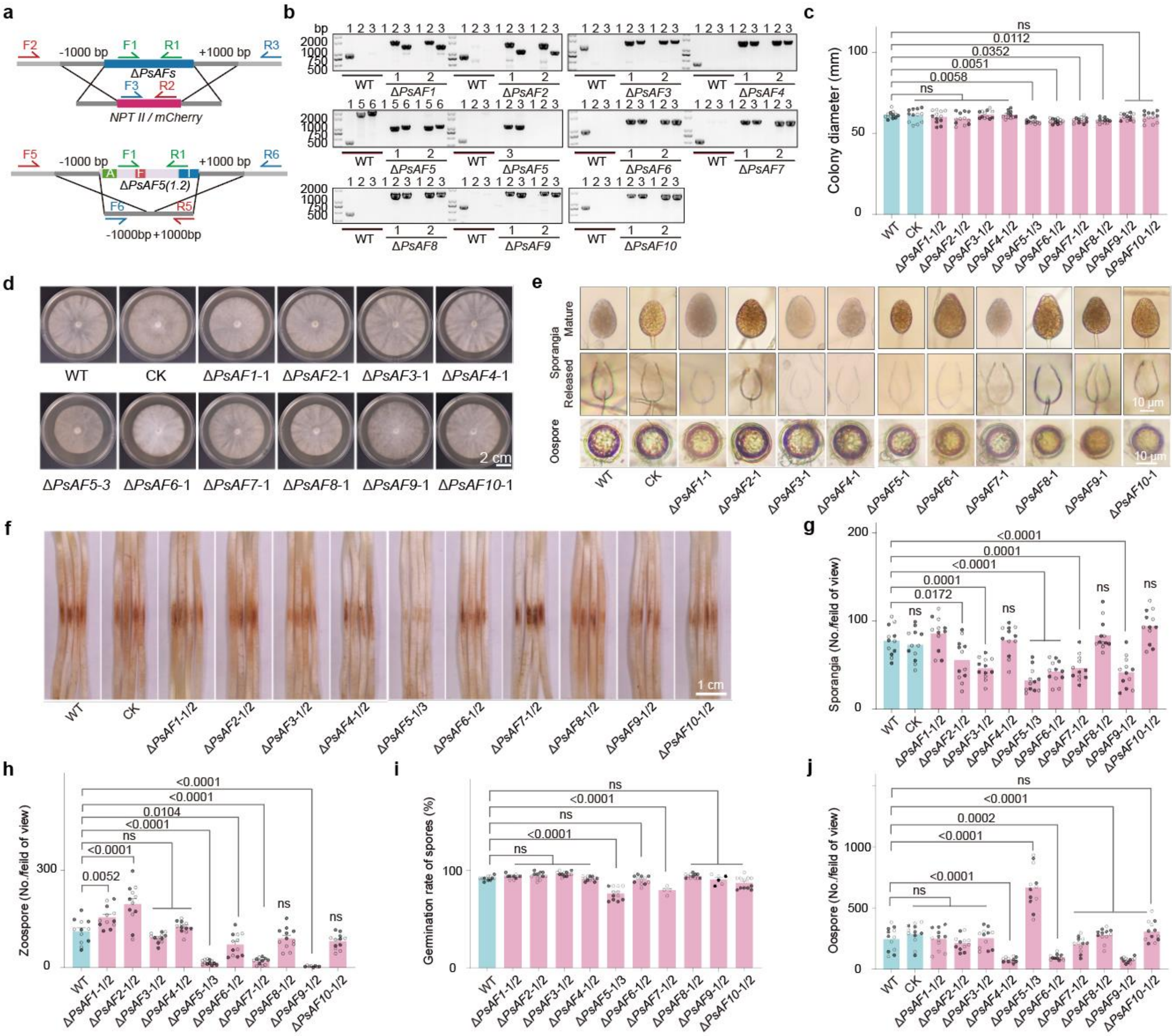

Supplementary Fig 2. The knockout pattern and phenotypes of Δ*PsAF* mutants. a Model of CRISPR/ Cas9-mediated knockout of *PsAF* genes knockout model. In strains Δ*PsAF1* to Δ*PsAF10*, the relevant *PsAF* gene was replaced with *NPT II* or *mCherry*, whereas in strains Δ*PsAF5-1 and* Δ*PsAF5-2* the *PsAF5* gene was directly deleted without replacement. Primer pairs for PCR verification are marked using red, green, and blue arrows. **b** PCR analysis of Δ*PsAF* mutants; the numbers above the lanes indicate that primer pairs were used at the positions labelled in (**a**). **c, d** Mycelia growth rate (**c**) and mycelial colony morphology (**d**) of the wild type, CK, and Δ*PsAF* mutants on V8 medium cultured at 25°C for 7 d. Scale bar, 2 cm. **e** Sporangia and oospore morphology of Δ*PsAF* mutants compared to wild type and CK strains. All strains were cultured on V8 medium at

25°C for 9 d. Scale bar, 10 μm. **f** Representative images of the hypocotyls of susceptible soybean (Williams) infected with the wild-type *P. sojae* strain, homozygous knockout mutants (Δ*PsAFs*), and a transformant without *PsAF* editing events (CK) at 36 h post-inoculation. Scale bars, 1 cm. **g-j** Sporangia number (**g**), zoospore number (**h**) and germination rate (**i**), and oospore number (**j**) of Δ*PsAF* mutants compared to wild type and CK strains. The solid and hollow circles represent two independent deletion mutants of each gene (n=12 biologically independent samples). All experiments in (**c-j**) were independently repeated twice with similar results. Data are presented as mean value ± SEM, ordinary one-way ANOVA and Dunnett's multiple comparisons test were used, and ns = non-significant. Source data are provided as a Source Data file.

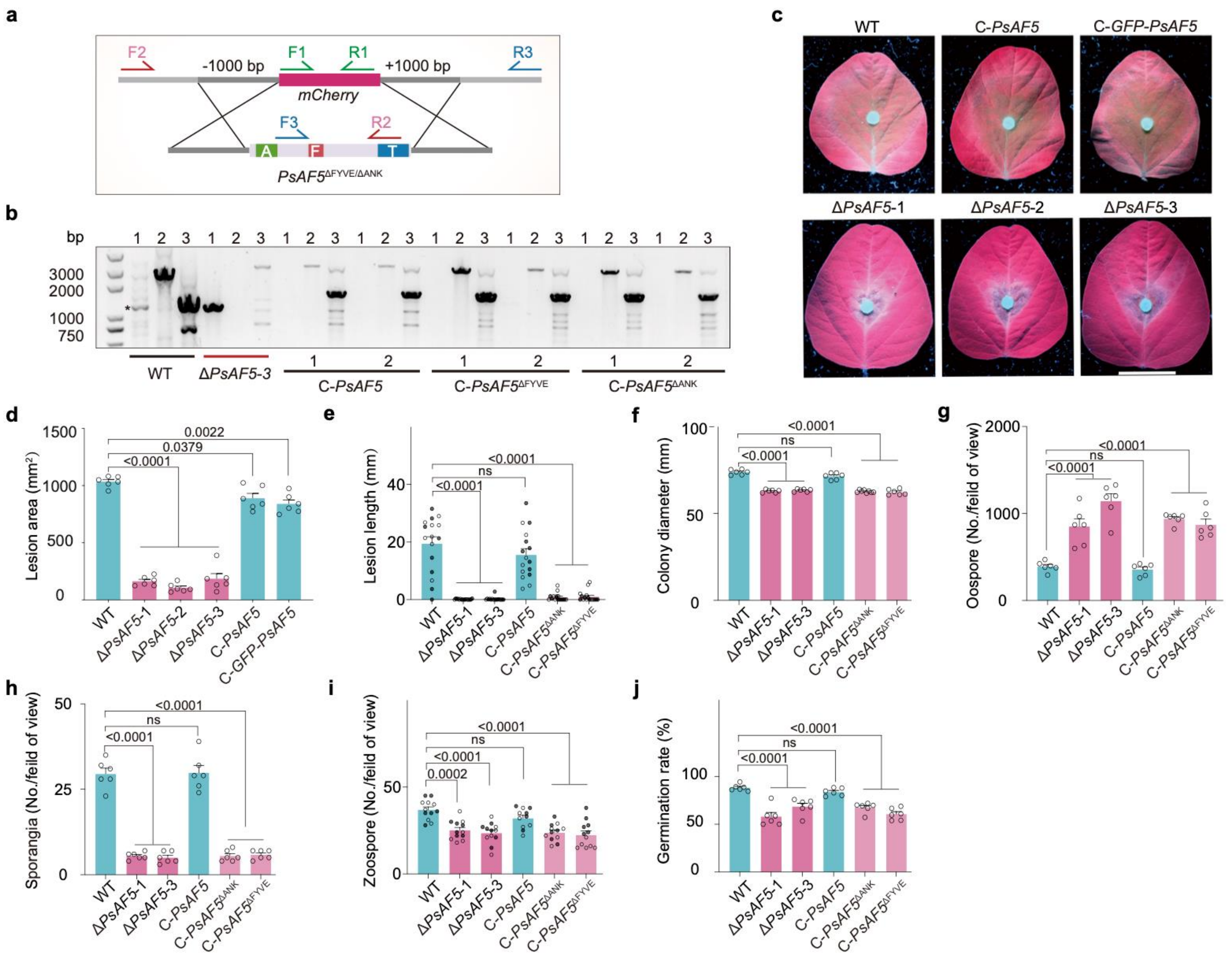

Supplementary Fig 3. Complementation of *PsAF5* knockout with wild type and mutant *PsAF5* genes. a Strategy for complementation of *PsAF5* knock out by CRISPR/Cas9-mediated replacement with various *PsAF5* genes. PCR verification primer pairs are marked using colored arrows. **b** PCR analysis of *PsAF5* full-length and domain truncated complement transformants; the numbers above the lanes indicate that primer pairs were used at the positions labelled in (**a**). **c, d** Infection of soybean unifoliate leaves by complemented transformants.

Representative images (**c**) and lesion areas (**d**) of soybean leaves 36 h post-inoculation with the wild type, Δ*PsAF5* mutants, and complemented transformants C-*PsAF5* and C-*GFP-PsAF5*. Scale bar, 2 cm. **e** Lesion lengths on soybean hypocotyls caused by complemented transformants. The disease symptoms were observed 2 d post-inoculation of the hypocotyls with wild type, Δ*PsAF5*, full-length *PsAF5* and ANK or FYVE domain truncated *PsAF5* complementary transformants. **f-j** The colony diameters (**f**), the numbers of oospores (**g**), numbers of sporangia (**h**), numbers of zoospores (**i**), and germination rates (**j**) of the wild-type P6497, Δ*PsAF5*, full length *PsAF5* and ANK or FYVE domain truncated *PsAF5* complementary transformant. Data in (**d, f, g, h, j**) with n=6 biologically independent samples; Data in (**e, i**) with n=16 or 12 biologically independent samples. All experiments in (**c-j**) were independently repeated twice with similar results. Data are presented as mean value ± SEM. Ordinary one-way ANOVA and Dunnett's multiple comparisons test were used, and ns = non-significant. Source data are provided as a Source Data file.

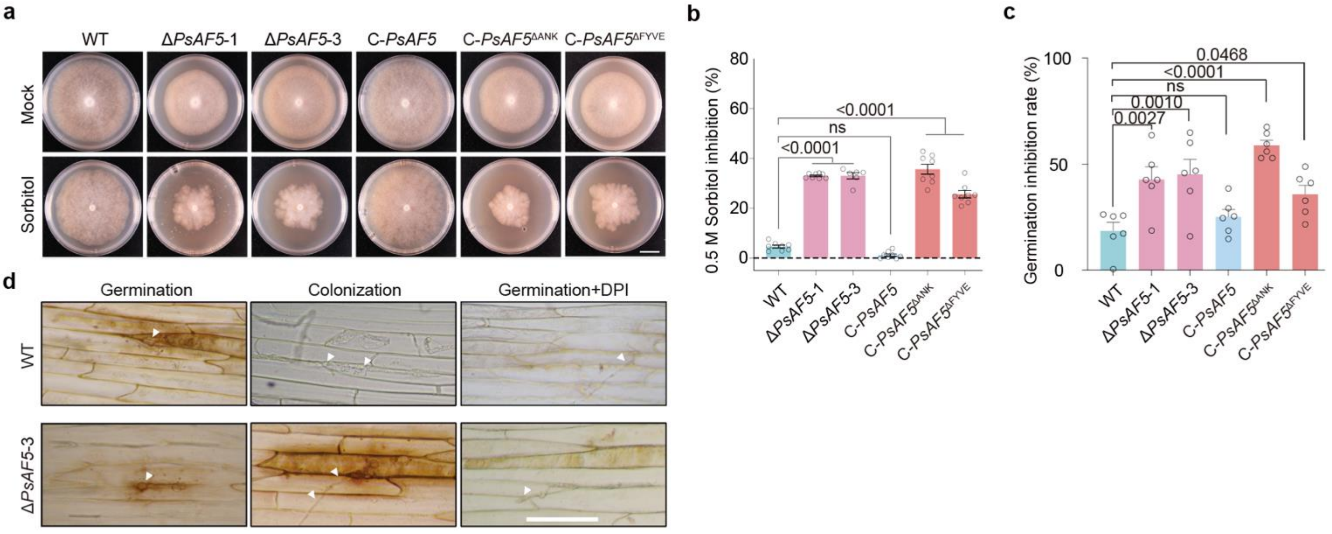

Supplementary Fig 4. Stress phenotypes of Δ*PsAF5* mutants and H_2O_2 accumulation during infection. a, b Partial or full deletions of *PsAF5* (wild type, Δ*PsAF5*, complemented transformants with full length *PsAF5* and ANK or FYVE domain deletions) increases the osmotic pressure sensitivity (0.5 M sorbitol) of mycelia. **a** colony morphology, **b** relative growth inhibition rates based on colony diameter (n=8 biologically independent samples). Ordinary one-way ANOVA and Dunnett's multiple comparisons test were used; ns = non-significant. **c** Inhibition of zoospore germination by 0.2 mM H_2O_2 (n=6 biologically independent samples). The data in (**b, c**) present as averages ± SEM. **d** DAB staining shows the accumulation of H_2O_2 in soybean hypocotyl cells after inoculation with zoospores of the wild type and Δ*PsAF5-3* mutant at the germination stage (2 h post-inoculation), colonization stage (6 h post-inoculation) or germination stage with DPI treatment (4 μM). White triangles indicate

the locations of the infecting germlings. All experiments were independently repeated twice with similar results. Scale bar, 50 μm. Source data are provided as a Source Data file.

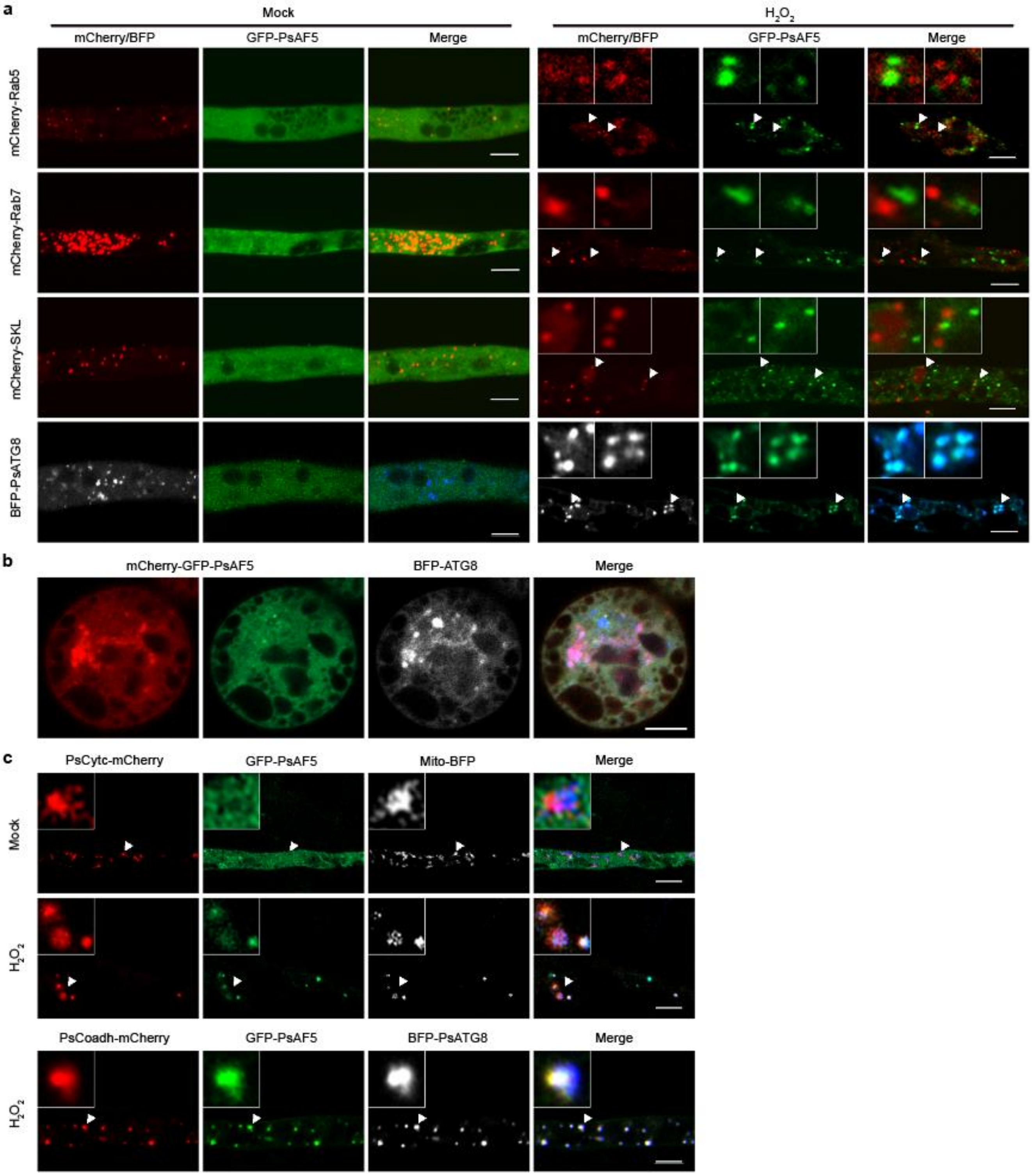

Supplementary Fig 5. Location of PsAF5 relative to different organelle markers. a Confocal images showing localization of GFP-PsAF5 relative to fluorescently-tagged markers for early endosomes (mCherry-PsRab5), late endosomes (mCherry-PsRab7), peroxisomes (mCherry-SKL C-terminal tripeptide), and autophagosomes (BFP-PsATG8) in *P. sojae*

transformants. Transformants were cultured in V8 liquid medium for 2 d then treated with or without H_2O_2 (1 mM, 1 h) as indicated. Scale bars, 5 μm. **b** Confocal images of BFP-ATG8 and mCherry-GFP-PsAF5 expressed in wild-type protoplasts, showing their localization in the cell. Scale bar, 5 μm. **c** Representative fluorescence images of PsCytc-mCherry, GFP-PsAF5 and Mito-BFP (mitochondria signal peptide fused to BFP label) in the wild type *P. sojae*, with or without 1 mM H_2O_2 treatment for 1 h. Mitochondrial matrix protein PsCoadh-mCherry, co-expressed with GFP-PsAF5 and BFP-PsATG8 in *P. sojae* treated with 1 mM H_2O_2 for 1 h was also visualized. All experiments were independently repeated 3 times with similar results. Scale bars, 5 μm.

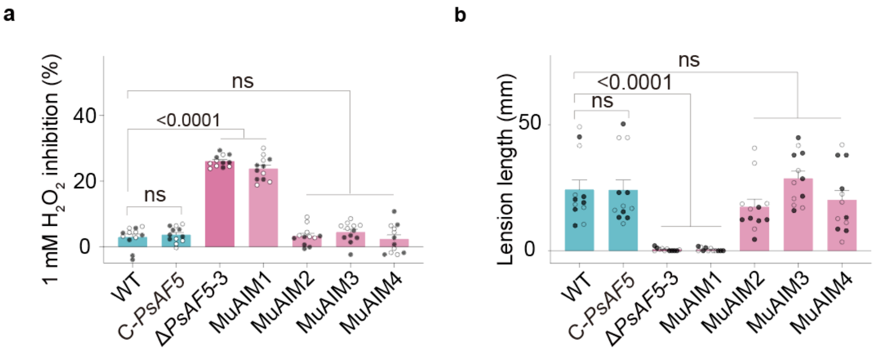

Supplementary Fig 6. H_2O_2 sensitivity and virulence of transformants with point mutations in the AIM motifs of PsAF5. a, b 1 mM H_2O_2 sensitivity (**a**) and virulence (**b**) of different AIM mutants of *PsAF5* (MuAIM1-4). The results were derived from two (n=12 biologically independent samples) biological replicates in each case. All experiments were independently repeated twice with similar results. Data are presented as mean value ± SEM. Ordinary one-way ANOVA and Dunnett's multiple comparisons test were used; ns = non-significant. Source data are provided as a Source Data file.

a

Serial number	mCherry-tag	GFP-tag	BFP-tag	Strain
①	mCherry-PsRab5	GFP-PsAF5	—	P6497
②	mCherry-PsRab7	GFP-PsAF5	—	P6497
③	mCherry-SKL	GFP-PsAF5	—	P6497
④	Mito-mCherry-GFP		BFP-PsATG8	P6497
⑤	Mito-mCherry-GFP		BFP-PsATG8	*ΔPsAF5-3*
⑥	mCherry-PsAF5	Mito-GFP	BFP-PsATG8	P6497
⑦	mCherry-PsAF5	—	BFP-PsATG8	P6497
⑧	mCherry-GFP-PsAF5		BFP-PsATG8	*ΔPsAF5-3*
⑨	mCherry-GFP-PsAF5-AIM1		BFP-PsATG8	*ΔPsAF5-3*
⑩	mCherry-GFP-PsAF5-AIM2		BFP-PsATG8	*ΔPsAF5-3*
⑪	mCherry-GFP-PsAF5-AIM3		BFP-PsATG8	*ΔPsAF5-3*
⑫	mCherry-GFP-PsAF5-AIM4		BFP-PsATG8	*ΔPsAF5-3*
⑬	mCherry-GFP-PsAF5-ANK		BFP-PsATG8	*ΔPsAF5-3*
⑭	mCherry-GFP-PsAF5-FYVE		BFP-PsATG8	*ΔPsAF5-3*
⑮	mCherry-GFP-Myc		BFP-PsATG8	P6497
⑯	Mito-mCherry-GFP		BFP-PsATG8	*ΔPsATG7*

b

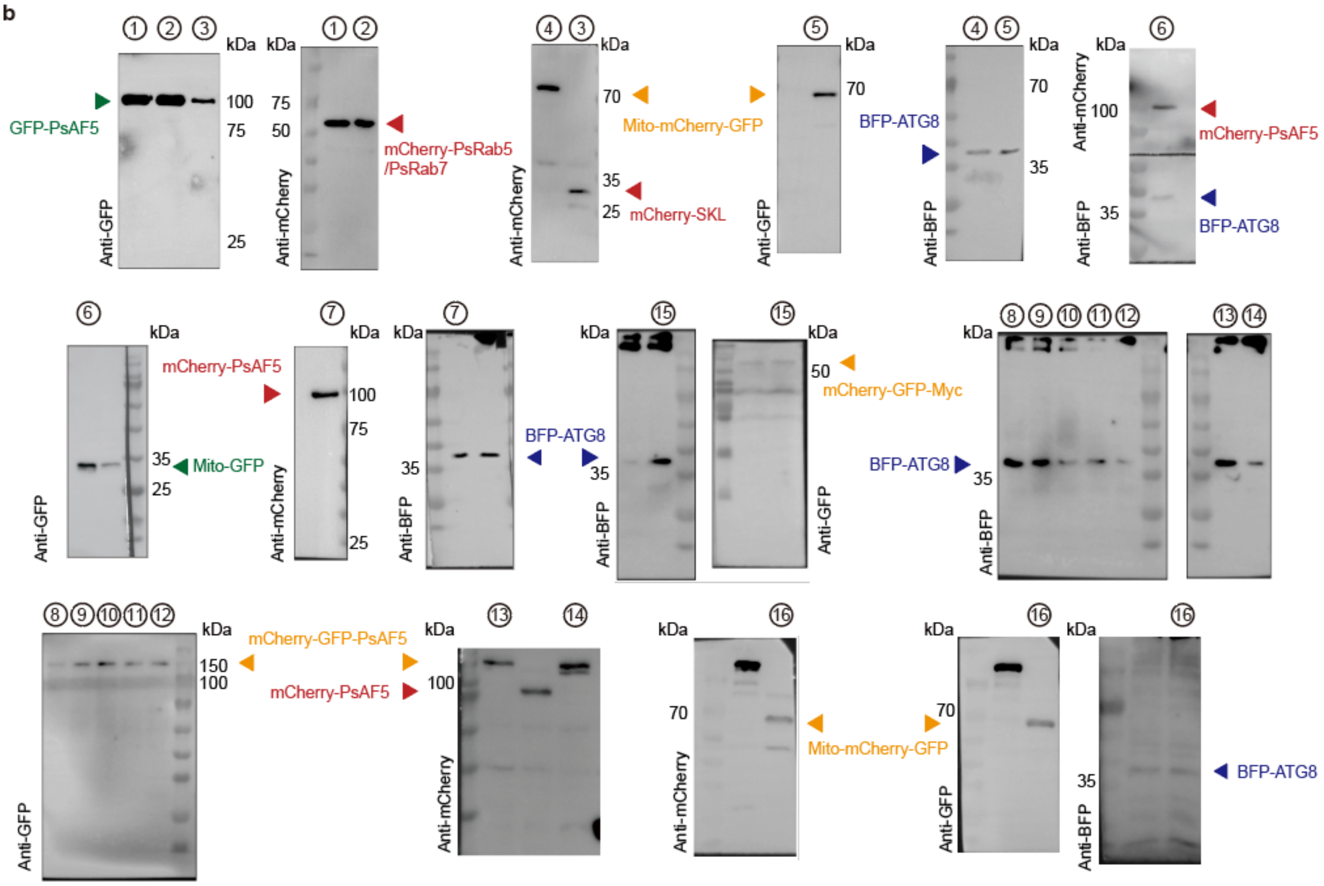

Supplementary Fig 7. Confirmation of protein expression in fluorescent transformants through immunoblotting. a The fluorescent transformants in the main figure and their serial number. **b** Immunoblot analysis revealed the expression of the fluorescently-tagged fusion protein corresponding to the described transformants in (**a**).

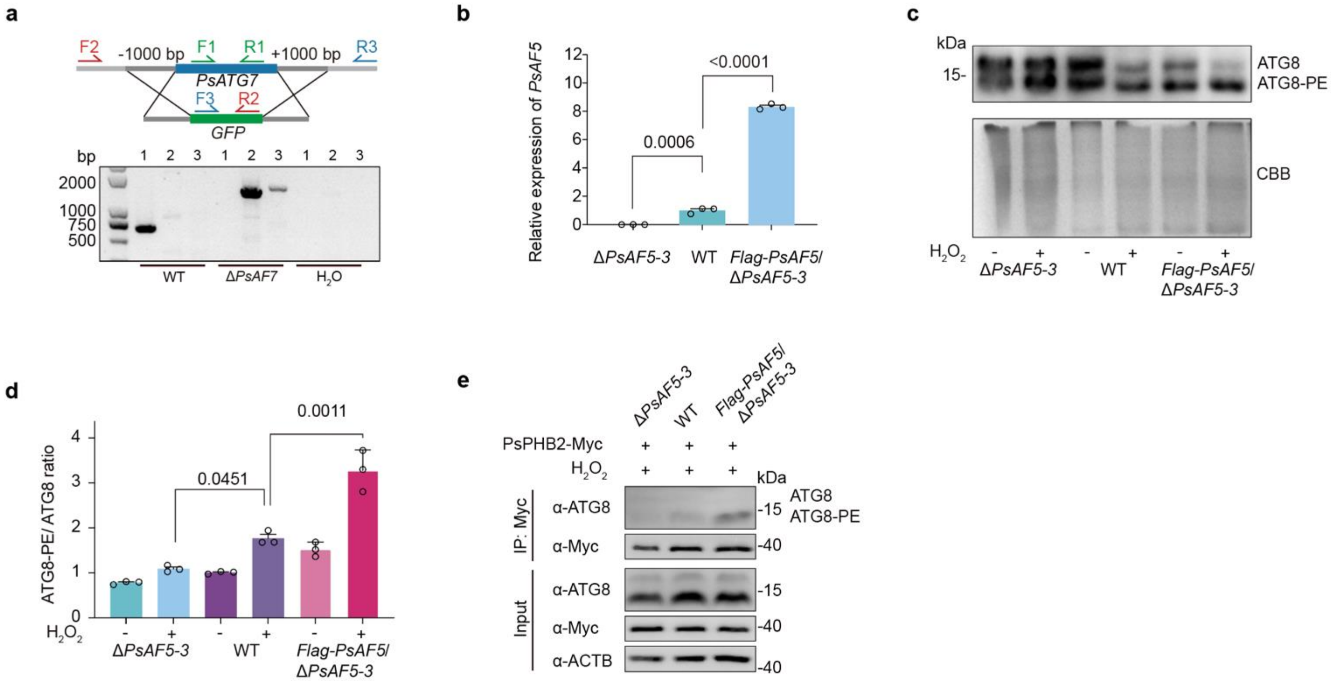

Supplementary Fig 8. Knockout of PsATG7, activation of PsATG8 in mitochondria, and interaction between PsATG8 and PsPHB2 after overexpression of Flag-PsAF5

a Knockout of CRISPR/ Cas9-mediated *PsATG7*. The *PsATG7* gene was replaced with *GFP*. Primer pairs for PCR verification are marked using red, green, and blue arrows. Lower panel represents PCR analysis of Δ*PsATG7* mutant. **b** qPCR results of *PsAF5* expression in wild type strain, *PsAF5* knockout and overexpression transformant. **c** The mitochondrial ATG8 lipidation ratio from purified mitochondria extracted from Δ*PsAF5-3*, the wild type and the Δ*PsAF5-3* transformant overexpressing *Flag-PsAF5*, treated with or without 1 mM H_2O_2 for 1 h. ATG8 lipidation was detected using ATG8 antibodies. **d** The ratio of lipidated to non-lipidated ATG8 was based on the gray value of the immunoblot bands shown in (**c**). Ordinary one-way ANOVA and Dunnett's multiple comparisons test were used; ns = non-significant. **e** Co-immunoprecipitation experiments showing that the levels of interaction between PsPHB2-Myc and PsATG8-PE were enhanced by the overexpression of *PsAF5*. 1 mM H_2O_2 was added 1 h before protein extraction. The proteins detected by the ATG8 antibody, in (**c**) and (**e**), were isolated using urea SDS–PAGE. All experiments were independently repeated twice with similar results. Source data are provided as a Source Data file.

Supplementary Table 1. Transcript levels of the *PsAF* genes in wild-type *P. sojae*

Gene Name	mycelia	sporulating hyphae	zoospore	Cysts	Cysts germination
PsAF1	0.006098	0.005712	0.009156	0.007266	0.004625
PsAF2	0.021515	0.017979	0.073186	0.069257	0.014039
PsAF3	0.225298	0.189242	0.051585	0.055196	0.038871
PsAF4	0.006252	0.007011	0.034856	0.032742	0.006347
PsAF5	1.000000	0.771969	1.101025	2.103756	2.130917
PsAF6	1.000000	0.775447	0.031327	0.172844	1.774307
PsAF7	0.333406	0.393726	0.092961	1.000000	0.407870
PsAF8	0.009921	0.005993	0.007341	0.029615	0.020717
PsAF9	0.399916	0.474642	0.024118	0.041539	0.092714
PsAF10	0.040265	0.055922	0.003406	0.015514	0.018247

Continues upper table

Gene Name	1.5 hpi*	3 hpi*	6 hpi*	12 hpi*	24 hpi*	48 hpi*
PsAF1	0.016442	0.004759	0.006828	0.004333	0.004543	0.002685
PsAF2	0.020700	0.013480	0.019550	0.026556	0.020198	0.016375
PsAF3	0.136422	0.138449	0.072229	0.069464	0.033269	0.028845
PsAF4	0.004363	0.004093	0.005844	0.006070	0.012934	0.008714
PsAF5	0.504622	0.232536	0.329381	0.387457	0.730849	0.934217
PsAF6	1.882722	1.000000	1.403955	1.039115	1.882500	1.440384
PsAF7	0.170395	0.183412	0.122953	0.121447	0.238507	0.120656
PsAF8	0.013699	0.008188	0.016526	0.008388	0.019516	0.009655

PsAF9	0.122664	0.067635	0.052372	0.168655	0.139069	0.190673
PsAF10	0.030382	0.011037	0.012809	0.022742	0.022609	0.022748

(Hpi: hours post-inoculation)

Supplementary Table 2. Primers used to generate donor vector construction of *PsAFs*

Primer Name	Primer Don1F	Primer Don1R	Primer Don2F	Primer Don2R	Primer Don3F	Primer Don3R
PsAF1	GATAAGCTTGATATCGAATTCTGTCCTACACGCTGGAGC	GTTCAATCATGTCTGTATGTCTGCTCTAGGG	ACATACAGACATGATTGAACAAGATGGATTGCACG	GGACATGTCCTCAGAAGAACTCGTCAAGAAGGC	GTTCTTCTGAGGACATGTCCTCGGCACC	CGCTCTAGAACTAGTGGATCCTTCCAACCTGGGCAGTCG
PsAF2	GATAAGCTTGATATCGAATTCGCGTGCTCACTCGGTCAA	GTTCAATCATCGTAGACGACGACGCTGCA	GTCGTCTACGATGATTGAACAAGATGGATTGCACG	AAGCCGCTCTTCAGAAGAACTCGTCAAGAAGGC	GTTCTTCTGAAGAGCGGCTTCGAGTGAA	CGCTCTAGAACTAGTGGATCCGCGGCGGCGCTGTGGAGT
PsAF3	GTATCGATAAGCTTGATATCGAATTCGGGCGAATATTTTGTTTTTCTGC	GTTCAATCATGACGGCGCGTAAGACG	ACGCGCCGTCATGATTGAACAAGATGGATTGCAC	ACGTCCCAGGTCAGAAGAACTCGTCAAGAAGGC	GTTCTTCTGACCTGGGACGTCCCTCG	GCGGCCGCTCTAGAACTAGTGGATCCTATTTTGGTGTTGTTTGTCGTTGG
PsAF4	GATAAGCTTGATATCGAATTCGGCTCCGGCGCTCGGTGC	GTTCAATCATGGTGGGGGAATGCTGTCC	TTCCCCCACCATGATTGAACAAGATGGATTGCACG	CGCCCGCCATTCAGAAGAACTCGTCAAGAAGGC	GTTCTTCTGAATGGCGGGCGGGGCATTTCATGC	CGCTCTAGAACTAGTGGATCCTGAGCCCCGGCAAGGGGG
PsAF5	GATAAGCTTGATATCGAATTCCTGGCG	GTTCAATCATGCTTCCTGTGTGTCTCTGTGTG	CACAGGAAGCATGATTGAACAAGATGGATTGCACG	GGAAGGCGCCTCAGAAGAACTCGTCAAGAAGGC	GTTCTTCTGAGGCGCCTTCCTCACAGGG	CGCTCTAGAACTAGTGGATCCAGCGTT

	TACCTCGTGTCG					CGTGGGGTTCTCG
PsAF5-MC	GATAAGCTTGATATCGAATTCCTGGCGTACCTCGTGTCG	TTGCTCACCATGCTTCCTGTGTGTCTCTGTGTG	ACACAGGAAGCATGGTGAGCAAGGGCGAGG	AGGAAGGCGCCTTACTTGTACAGCTCGTCCATGC	GTACAAGTAAGGCGCCTTCCTCACAGGG	CGCTCTAGAACTAGTGGATCCAGCGTTCGTGGGGTTCTCG
PsAF6	GATAAGCTTGATATCGAATTCGTATCTGTGTTGCGGAAGGC	GTTCAATCATGGCTTGGGTCGTTTCCGG	GACCCAAGCCATGATTGAACAAGATGGATTGCACG	CTTGCCGTGTTCAGAAGAACTCGTCAAGAAGGC	GTTCTTCTGAACACGGCAAGCATCAAGG	CGCTCTAGAACTAGTGGATCCGGACACGGAGTTGACTCT
PsAF7	GATAAGCTTGATATCGAATTCGTCTCAGCCAAGCAGGAGTG	GTTCAATCATGCGCGCAGAGAATGGCTG	CTCTGCGCGCATGATTGAACAAGATGGATTGCACG	CTGGTTCTACTCAGAAGAACTCGTCAAGAAGGC	GTTCTTCTGAGTAGAACCAGAAGGCCTTCAGTC	CGCTCTAGAACTAGTGGATCCGTGCAATGGGAGACGCAC
PsAF8	GATAAGCTTGATATCGAATTCATGCCTGAATCAACTTGACAATGCC	GTTCAATCATCGCTGCTGGCACGGAGGA	GCCAGCAGCGATGATTGAACAAGATGGATTGCACG	GTAGTCGCGCTCAGAAGAACTCGTCAAGAAGGC	GTTCTTCTGAGCGCGACTACTGTCGTTGGA	CGCTCTAGAACTAGTGGATCCTAAGAAGCTGAAGTCGACATCAGA
PsAF9	GATAAGCTTGATATCGAATTCTGGCAGGAGGACGCAACA	GTTCAATCATAGTGTCTGTCTCTGTGTGGGGA	GACAGACACTATGATTGAACAAGATGGATTGCACG	TCGCGCGAGTTCAGAAGAACTCGTCAAGAAGGC	CGAGTTCTTCTGAACTCGCGCGAACACTG	CGCTCTAGAACTAGTGGATCCCGCTCTGCCTCCCGGAGACC

PsAF10	GATAAGCTTGATATCGAATTCGGCGTTTTCCCGACGGGAGC	GTTCAATCATGCTCATTAGTTCCTTCCGCCG	ACTAATGAGCATGATTGAACAAGATGGATTGCACG	CAGTGATCTTTCAGAAGAACTCGTCAAGAAGGC	GTTCTTCTGAAAGATCACTGGCAAGTCAGAC	CGCTCTAGAACTAGTGGATCCTGAAAGCCAAGGGCTGCAC
PsATG7	ATCGATAAGCTTGATATCGAATTCATTCCGAATTGGTCCGCTATTTTC	CCTCGCCCTTGCCCATGGTCTCTTCGGTTTGAAGAATTCTTC	CTTCAAACCGAAGAGACCATGGGCAAGGGCGAGG	GTATCGGCAGACCACGCTCAACGCGTTCCGGAG	CTCCGGAACGCGTTGAGCGTGGTCTGCCGATAC	GCGGCCGCTCTAGAACTAGTGGATCCGCCCACATGTACCCGTTATCG

Supplementary Table 3. sgRNA sequence for *PsAFs* and *mCherry* genes

Primer Name	Positive-sense strand	Negative-sense strand
PsAF1-sg1	CTAGCGGGCATCTGATGAGTCCGTGAGGACGAAACGAGTAAGCTCGTCATGCCCACGACCTCGTACTA	AAACTAGTACGAGGTCGTGGGCATGACGAGCTTACTCGTTTCGTCCTCACGGACTCATCAGATGCCCG
PsAF1-sg2	CTAGCAGTGGACTGATGAGTCCGTGAGGACGAAACGAGTAAGCTCGTCTCCACTGTACGTAAGCTCGT	AAACACGAGCTTACGTACAGTGGAGACGAGCTTACTCGTTTCGTCCTCACGGACTCATCAGTCCACTG
PsAF2-sg1	CTAGCGTTGACCTGATGAGTCCGTGAGGACGAAACGAGTAAGCTCGTCGTCAACCTGCCAGATCGCAA	AAACTTGCGATCTGGCAGGTTGACGACGAGCTTACTCGTTTCGTCCTCACGGACTCATCAGGTCAACG
PsAF2-sg2	CTAGCACGGCCCTGATGAGTCCGTGAGGACGAAACGAGTAAGCTCGTCGGCCGTCGTGACCTCGCTAC	AAACGTAGCGAGGTCACGACGGCCGACGAGCTTACTCGTTTCGTCCTCACGGACTCATCAGGGCCGTG

PsAF3-sg1	CTAGCATCTTCCTGATGAGTCCGTGAGGACGAAACGAGTAAGCTCGTCGAAGATGCTCATGGCCAAAG	AAACCTTTGGCCATGAGCATCTTCGACGAGCTTACTCGTTTCGTCCTCACGGACTCATCAGGAAGATG
PsAF3-sg2	CTAGCACGCGCCTGATGAGTCCGTGAGGACGAAACGAGTAAGCTCGTCGCGCGTTGCCCATCTCTGTC	AAACGACAGAGATGGGCAACGCGCGACGAGCTTACTCGTTTCGTCCTCACGGACTCATCAGGCGCGTG
PsAF4-sg1	CTAGCGTCAGACTGATGAGTCCGTGAGGACGAAACGAGTAAGCTCGTCTCTGACCAGGCACCGCGATG	AAACCATCGCGGTGCCTGGTCAGAGACGAGCTTACTCGTTTCGTCCTCACGGACTCATCAGTCTGACG
PsAF4-sg2	CTAGCGTCCAACTGATGAGTCCGTGAGGACGAAACGAGTAAGCTCGTCTTGGACATGAGCGACATATC	AAACGATATGTCGCTCATGTCCAAGACGAGCTTACTCGTTTCGTCCTCACGGACTCATCAGTTGGACG
PsAF5-sg1	CTAGCATATCACTGATGAGTCCGTGAGGACGAAACGAGTAAGCTCGTCTGATATTAACCGCCTCTCCT	AAACAGGAGAGGCGGTTAATATCAGACGAGCTTACTCGTTTCGTCCTCACGGACTCATCAGTGATATG
PsAF5-sg2	CTAGCGAAGGCCTGATGAGTCCGTGAGGACGAAACGAGTAAGCTCGTCGCCTTCTTGAACCAACCCGG	AAACCCGGGTTGGTTCAAGAAGGCGACGAGCTTACTCGTTTCGTCCTCACGGACTCATCAGGCCTTCG
PsAF6-sg1	CTAGCGTAGGCCTGATGAGTCCGTGAGGACGAAACGAGTAAGCTCGTCGCCTACCCGTACATTGACAC	AAACGTGTCAATGTACGGGTAGGCGACGAGCTTACTCGTTTCGTCCTCACGGACTCATCAGGCCTACG
PsAF6-sg2	CTAGCAGCGGCCTGATGAGTCCGTGAGGACGAAACGAGTAAGCTCGTCGCCGCTGTGCTCCAATTCCA	AAACTGGAATTGGAGCACAGCGGCGACGAGCTTACTCGTTTCGTCCTCACGGACTCATCAGGCCGCTG
PsAF7-sg1	CTAGCCAACACCTGATGAGTCCGTGAGGACGAAACGAGTAAGCTCGTCGTGTTGCTGCTGAAAACCGG	AAACCCGGTTTTCAGCAGCAACACGACGAGCTTACTCGTTTCGTCCTCACGGACTCATCAGGTGTTGG

PsAF7-sg2	CTAGCTTGCCTCTGATGAGTCCGTGAGGACGAAACGAGTAAGCTCGTCAGGCAACGGCTGAAGTACGA	AAACTCGTACTTCAGCCGTTGCCTGACGAGCTTACTCGTTTCGTCCTCACGGACTCATCAGAGGCAAG
PsAF8-sg1	CTAGCCAATACCTGATGAGTCCGTGAGGACGAAACGAGTAAGCTCGTCGTATTGCCGTCGTTCTTCCG	AAACCGGAAGAACGACGGCAATACGACGAGCTTACTCGTTTCGTCCTCACGGACTCATCAGGTATTGG
PsAF8-sg2	CTAGCCTGTCCCTGATGAGTCCGTGAGGACGAAACGAGTAAGCTCGTCGGACAGCGACCACCAGACCG	AAACCGGTCTGGTGGTCGCTGTCCGACGAGCTTACTCGTTTCGTCCTCACGGACTCATCAGGGACAGG
PsAF9-sg1	CTAGCCCTGAACTGATGAGTCCGTGAGGACGAAACGAGTAAGCTCGTCTTCAGGTGCTCGGACAGCCG	AAACCGGCTGTCCGAGCACCTGAAGACGAGCTTACTCGTTTCGTCCTCACGGACTCATCAGTTCAGGG
PsAF9-sg2	CTAGCGGACAGCTGATGAGTCCGTGAGGACGAAACGAGTAAGCTCGTCCTGTCCGAGCACCTGAACGC	AAACGCGTTCAGGTGCTCGGACAGGACGAGCTTACTCGTTTCGTCCTCACGGACTCATCAGCTGTCCG
PsAF10-sg1	CTAGCAACAGCCTGATGAGTCCGTGAGGACGAAACGAGTAAGCTCGTCGCTGTTCCTCAAAAGTATGC	AAACGCATACTTTTGAGGAACAGCGACGAGCTTACTCGTTTCGTCCTCACGGACTCATCAGGCTGTTG
PsAF10-sg2	CTAGCCCGGCCCTGATGAGTCCGTGAGGACGAAACGAGTAAGCTCGTCGGCCGGAACTGACCCCAACA	AAACTGTTGGGGTCAGTTCCGGCCGACGAGCTTACTCGTTTCGTCCTCACGGACTCATCAGGGCCGGG
mCherry-sg1	CTAGCTACACCCTGATGAGTCCGTGAGGACGAAACGAGTAAGCTCGTCGGTGTAGTCCTCGTTGTGGG	AAACCCCACAACGAGGACTACACCGACGAGCTTACTCGTTTCGTCCTCACGGACTCATCAGGGTGTAG
mCherry-sg2	CTAGCACCATGCTGATGAGTCCGTGAGGACGAAACGAGTAAGCTCGTCCATGGTCTTCTTCTGCATTA	AAACTAATGCAGAAGAAGACCATGGACGAGCTTACTCGTTTCGTCCTCACGGACTCATCAGCATGGTG

PsATG7-sg1	CTAGCAGCATCCTGATGAGTCCGTGAGGACGAAACGAGTAAGCTCGTCGATGCTGAGAAGCCTAGCGA	AAACTCGCTAGGCTTCTCAGCATCGACGAGCTTACTCGTTTCGTCCTCACGGACTCATCAGGATGCTG
PsATG7-sg2	CTAGCCACCGCCTGATGAGTCCGTGAGGACGAAACGAGTAAGCTCGTCGCGGTGCTACATTCCCCTCA	AAACTGAGGGGAATGTAGCACCGCGACGAGCTTACTCGTTTCGTCCTCACGGACTCATCAGGCGGTGG

Supplementary Table 4. Primers used for PCR verification of transformants

**Primer Name**	**Primer Sequences (5' to 3')**	**Description**
PsAF1-F1	CGAAGGGCTGATGAACCAC	To validate *PsAF1* gene knockout transformants.
PsAF1-R1	CGCTCGCTAGAGGATCAATG	
PsAF1-F2	TGGCTGAAGTTGCGACTGAAG	
NPT2-R2	GAAGGCGATAGAAGGCGATG	
NPT2-F3	GCATCGCCTTCTATCGCC	
PsAF1-R3	ACCTACAACGCACACCAAC	
PsAF2-F1	CCGTCGTGACCTCGCTAC	To validate *PsAF2* gene knockout transformants.
PsAF2-R1	TCCCTCTCGTCTCCCTCC	
PsAF2-F2	GACGACGCCGCAGAGA	
NPT2-R2’	GGAGCAAGGTGAGATGACAG	
NPT2-F3’	GCCTTCTATCGCCTTCTTGAC	
PsAF2-R3	CGTTGCCTGGACCATTGC	
PsAF3-F1	ATGCCCTCCGCCGAA	To validate *PsAF3* gene knockout transformants.
PsAF3-R1	TTATGCCGTCTCGCTGTGT	
PsAF3-F2	CTAAGACGAGCTACGGGTCG	
NPT2-R2“	GCTCTTCGTCCAGATCATCC	

NPT2-F3''	CCTGTCATCTCACCTTGCT	
PsAF3-R3	ACACATACGTTCGCCCTC	
PsAF4-F1	TCGTGGTCAAGGTGGTCTTC	To validate *PsAF4* gene knockout transformants.<br>Use NPT2-F3/R2'' primers.
PsAF4-R1	ATCTGGTAGTGTCGGTGGT	
PsAF4-F2	GCATTTGGGTGGAGTGAGC	
PsAF4-R3	TTCTGCTCTAAGCGTGGGC	
PsAF5-F1	AGACGACGAGAACCACAACAC	To validate *PsAF5* gene knockout transformants.
PsAF5-R1	GCTTAATGCCTCACGGAACAC	
PsAF5-F2/5	TTCCACTGAAGTAGCTGGCG	
mCherry-R2	CACCTTGAAGCGCATGAACT	
mCherry-F3	ACTACACCATCGTGGAACAGTAC	
PsAF5-F6	CACAGAGACACACAGGAAGC	
PsAF5-R5	CTCGTGCTTCATGGGTGC	
PsAF5-R3/6	CGTCTTGGACACCACGGG	
ZA6'555F	GACCAGCAACTTCAGCAGAG	To validate *PsAF6* gene knockout transformants.<br>Use NPT2-F3/R2'' primers.
ZA6'1511R	TCGGCAACAGCATTCAACAC	
ZA6-1055F	TTCCGACCCAAACTCCCTG	
ZA6+1056R	TTCAGACCGGATGCTGGGA	
ZA7'1148F	CGAGGATGAGAACGATGAGAAC	To validate *PsAF7* gene knockout transformants.<br>Use NPT2-F3/R2'' primers.
ZA7'1727R	AACAAGGCAACGGCTGAAG	
ZA7-1055F	CTACGGATCTACTGCGGC	
ZA7+1087R	GCTTCGAGGACTTGACCACC	
ZA8'453F	ATCCACATCGCCGTCAAG	

ZA8'932R	GTATTGCCGTCGTTCTTCC	To validate *PsAF8* gene knockout transformants.<br>Use NPT2-F3/R2'' primers.
ZA8-1080F	GAGAATGAGTCAATGCGGGC	
ZA8+1093R	TCCGTGTGCAAGTCGGC	
ZA9'162F	TTCAGCGTCACGTCGTCC	To validate *PsAF9* gene knockout transformants.<br>Use NPT2-F3/R2'' primers.
ZA9'849R	GTTGCGGCAGATCTTGCA	
ZA9-1137F	TTTCATCGTACGCTGCCAGC	
ZA9+1044R	TACATGGTGATCGCCATGGAC	
ZA10'329F	CATCTCGTCCGTGCTGC	To validate *PsAF10* gene knockout transformants.<br>Use NPT2-F3/R2'' primers.
ZA10'1153R	CCTGCTTCCTCTTGCGTTC	
ZA10-1128F	TCGAGTTGTAAGACGTCGTGG	
ZA10+1117R	TGCTACGTTACGACGGCG	
PsATG7-F1	AGCACGTCCACCAGTTTACTG	To validate *PsATG7* gene knockout transformants.
PsATG7-R1	CTCTCGCGCGAATCCG	
PsATG7-F2	ATGGAAGTATTGTGCTGTTACGG	
GFP-R2	GTTCACATCACCATCCAGTTCC	
GFP-F3	AACGAGAAGAGGGACCACA	
PsATG7-R3	GCAGTAGAAACTCCGCCAT	

Supplementary Table 5. Phenotypes of the *PsAF* knockout mutants in comparison with the control transformant.

**Strain**	**Growth rate***	**Virulence (Disease length, mm)**	**Sporangia number***	**Zoospore number***	**Oospore number***	**Germination rate (%)**
Δ*PsAF1*	-3.65%	8.07 (2.53)	44.15%	51.57%	3.33%	93.67 (2.23)
Δ*PsAF2*	1.52%	11.52 (3.57)	-4.62%	71.60%	-3.31%	94.50 (3.43)
Δ*PsAF3*	-0.06%	13.85 (6.31)	-32.12%	-1.87%	3.75%	96.50 (2.43)
Δ*PsAF4*	2.93%	10.23 (4.36)	8.64%	-3.81%	-35.10%	91.50 (2.28)
Δ*PsAF5*	-7.21%	2.19 (1.73)	-46.64%	-87.97%	298.40%	76.33 (6.54)
Δ*PsAF6*	-10.30%	8.73 (5.58)	-31.90%	-27.58%	-46.72%	90.17 (4.22)
Δ*PsAF7*	-8.47%	4.21 (1.93)	-30.98%	-78.48%	-25.95%	79.50 (4.12)
Δ*PsAF8*	-5.98%	18.66 (10.27)	29.15%	-17.41%	-1.94%	94.17 (2.17)
Δ*PsAF9*	-7.87%	7.81 (4.92)	-26.62%	-86.55%	-33.26%	90.67 (4.84)
Δ*PsAF10*	-7.51%	7.47 (3.79)	2.97%	-44.02%	4.49%	87.17 (6.12)
Control	0.00%	14.3 (7.37)	0.00%	0.00%	0.00%	92.00 (2.09)

The growth rate, virulence, sporangia number, zoospore number, oospore number, and germination rate were assayed with the control transformant and all the mutant strain.

The results for virulence and germination rate are presented as mean (SD).

* Percentage of differences compared with control transformant.

Supplementary Table 6. Phenotypes of various *PsAF5* mutants in comparison with the WT strain.

Strain	Colony diameter (mm)	1 mM H_2O_2 inhibition (%)	0.5 M sorbitol inhibition (%)	Lesion area (mm^2)	Lesion length (mm)	Oospore quantity	Sporangia quantity	Zoospore quantity	Zoospore germin-ation rate (%)	Germination inhibition rate (0.2 mM H_2O_2) (%)
WT	73.84 (1.47)	-1.82 (4.44)	4.61 (1.66)	1034 (47.22)	21.48 (8.51)	390.50 (58.96)	29.50 (4.14)	36.92 (5.71)	88.23 (3.44)	18.52 (9.94)
Δ*PsAF5*-1	62.93 (0.84)	26.59 (6.74)	33.01 (0.95)	163.3 (41.13)	0.11 (0.15)	851.50 (214.3)	5.50 (1.05)	25.17 (5.44)	58.09 (10.20)	42.88 (14.68)
Δ*PsAF5*-2	n.t	n.t	n.t	255.7 (64.98)	n.t	n.t	n.t	n.t	n.t	n.t
Δ*PsAF5*-3	63.36 (0.81)	27.49 (4.15)	33.03 (0.95)	185.0 (109.1)	0.24 (0.68)	1144.00 (201.20)	5.00 (1.67)	23.50 (6.02)	68.50 (8.25)	45.23 (17.52)
C-*PsAF5*	71.52 (1.92)	4.29 (2.91)	1.19 (1.42)	889.3 (102.4)	17.19 (7.83)	357.00 (66.67)	29.83 (5.27)	32.00 (5.75)	83.78 (3.93)	25.21 (8.36)
GFP-PsAF5/Δ*PsAF5*	n.t	n.t	n.t	839.8 (84.94)	n.t	n.t	n.t	n.t	n.t	n.t
C-*PsA*	62.73 (0.78)	30.69 (6.57)	35.69 (5.75)	n.t	0.88 (1.38)	940.80 (60.92)	5.50 (1.64)	26.11 (4.70)	67.44 (5.32)	58.93 (6.06)

F5^{ANK}										
C-*PsA* *F5*^{FYVE}	62.33 (1.67)	21.34 (10.42)	25.64 (4.08)	n.t	1.09 (1.90)	871.20 (155.10)	5.83 (1.33)	22.65 (6.72)	60.65 (6.19)	35.86 (10.29)
C-*PsA* *F5*^{TPR}	70.54 (2.41)	6.01 (2.47)	12.54 (3.95)	n.t	1.60 (3.03)	309.50 (25.86)	30.33 (3.08)	50.61 (8.40)	86.10 (2.43)	19.50 (3.98)

The growth rate, virulence, sporangia number, zoospore number, oospore number, and germination rate were assayed with the control transformant.

The results for virulence and germination rate are presented as mean (SD).

“n.t” means “not tested”.

Supplementary Table 7 Biomass assays of the virulence of *PsAF5* knockout and complementation transformants

Group	WT	Δ*PsAF5*-1	Δ*PsAF5*-2	Δ*PsAF5*-3	C-*PsAF5*
Mock treatment	0.040430	0.000186	0.000741	0.000273	0.056386
	0.043667	0.000202	0.000491	0.000282	0.041990
	0.040603	0.000238	0.000532	0.000288	0.049137
Diphenyleneiodonium (DPI) treatment	0.023305	0.027053	0.010024	0.014631	0.031272
	0.020795	0.020154	0.014093	0.017832	0.028183
	0.019517	0.024012	0.013310	0.017807	0.041252

Supplementary Table 8. Interacting proteins obtained by the IP-MS/MS method using PsAF5 as a baiting protein

Blast annotation of protein match	Location	PEP score	Unique Pep.
Apoptosis-inducing factor B	Mito.	57.713	2
Carbamoyl phosphate synthetase	Mito.	40.459	16
Acetyl-CoA Synthetase	Mito.	40.289	6
Prohibitin 1	Mito.	26.662	8
Homoserine acetyltransferase	Cyto.	24.836	9
Prohibitin 2	Mito.	22.044	9
Myosin	Cyto.	20.316	6
2-hydroxyacid dehydrogenase	Cyto.	19.602	5
Succinyl-CoA synthetase	Mito.	17.374	8
Cation-transporter ATPase	ER	15.967	8

Supplementary Table 9. Transcript levels of *PsAF5* in knockout and overexpression transformants

**Gene Name**	**WT**	**Δ*PsAF5***	**Flag-PsAF5/Δ*PsAF5***
PsAF5	1.114108	0	8.529832
	1.126280	0	8.271751
	0.759612	0.009583	8.148629

Transcript levels were normalized to the mean of WT.

Supplementary Table 10. Primers used to generate Fluorescence, Co-immunoprecipitation, and Pull-down constructs

Primer Name	Primer Sequences (5' to 3’)	Description
pYF3-GFP-F	GCCTCCGCGGACTAGTATGGGCAAGGGCGAGG	Construction of a *GFP-PsAF5* overexpression vector
GFP-AF5-R	TGGGCTTCATACGCGTTCCGGAGTTAAC	
GFP-AF5-F	CGGAACGCGTATGAAGCCCAAGGCCG	
AF5-pYF3-R	AAGAAGTAGGCACCGGTACCGGGCCCTTACCATCCAACAGTCATCGAGC	
pYF3-MC-F	TTATCGATAGGCCTCCGCGGACTAGTGCCACCatgGTGAGCAAGGGCGA	Construction of an *mCherry-PsAF5* overexpression vector
MC-GS-R	GCCACCGCCGCTGCCTCCTCCACCTCTAGACTTGTACAGCTCGTCCATG	
GS-AF5-F	GGAGGCAGCGGCGGTGGCGGTAGCGGATCCATGAAGCCCAAGGCCGC	
GS-AF5-F	GGAGGCAGCGGCGGTGGCGGTAGCGGATCCATGAAGCCCAAGGCCGC	Construction of an *mCherry-GFP-PsAF5* overexpression vector
MC-KoGFP-R	TTCCTCGCCCTTGCCCATGGTGGCCTTAATTAACTTGTACAGCTCGTCCATGC	
KoGFP-F	AGGCCACCATGGGCAAGGGCGAGG	
GFP-GS-R	GCCGCTGCCTCCTCCACCTCTAGAACGCGTTCCGGAGTTAAC	
GS-AF5 -R	CATGGATCCGCTACCGCCACCGCCGCTGCCTCCTCCACCtct	
pYF3-Flag-F	AGCTTATCGATAGGCCTCCGCGGACTAGTATGGACTACAAAGACGAT	Construction of a *Flag-PsAF5* overexpression vector

GS2-AF5-R	GTCCTGCGCGGCCTTGGGCTTCATGGATCCCGACCCACCTCCGCC	
GS2-AF5-F	CGGATCAGGCGGAGGTGGGTCGGGATCCATGAAGCCCAAGGCCG	
pYF3-ATPase-F	AAGCTTATCGATAGGCCTCCGCGGACTAGTGCCACCATGCTGTCTCGTGTGGGTCTTA	Construction of a mitochondrial localization marker vector labeled with mCherry.
ATPase-GS-R	CCTCCTCCACCGGATCCAATGGGCGGCAAGTTGTC	
ATPase-GS-MC-F	TTCAAGGACAACTTGCCGCCCATTGGATCCGGTGGAGGAGGCAGCGGCGGTGGCGGTAGCATGGTGAGCAAGGGCG	
MC-pYF3-R	AAGAAGTAGGCACCGGTACCGGGCCCCTACTTGTACAGCTCGTCCATG	
ATPase-GS-GFP-F	ATTGGATCCGGTGGAGGAGGCAGCGGCGGTGGCGGTAGCTTAATTAAGATGGGCAAGGGCGAGG	Construction of a mitochondrial localization marker vector labeled with GFP.
GFP-pYF3-R	TTACAAGAAGTAGGCACCGGTACCGGGCCCTTAACGCGTTCCGGAGTTAAC	
GS-PsRab5-F	GCAGCGGCGGTGGCGGTAGCGGATCCATGTCGCAGGGAAAGACGT	Construction of an *mCherry-Rab5* overexpression vector
PsRab5-pYF3-R	AGAAGTAGGCACCGGTACCGGGCCCCTAGCAGCATCCGCTCTTCG	
GS-PsRab7-F	GCAGCGGCGGTGGCGGTAGCGGATCCATGTCGCACCGCAAGAAA	Construction of an *mCherry-Rab7* overexpression vector
PsRab7-pYF3-R	AGAAGTAGGCACCGGTACCGGGCCCTTAACAGCATGAAGAGCTTTCGCG	

GS-SKL-pYF3-R	AAGAAGTAGGCACCGGTACCGGCCCTTACAGCTTCGATGCGGCCGCGCTACCGCCACCGCCGCTGC	Construction of an *mCherry-SKL* overexpression vector
pYF3-KoBFP-F	AAGCTTATCGATAGGCCTCCGCGGACTAGTGCCACCATGAGCGAGC	Construction of a *BFP-ATG8* overexpression vector
pmdc GS-ATG8-R	GAATGAGCTCATACTAGTGCTACCGCCACC	
pmdc GS-ATG8-F	GGTAGCACTAGTATGAGCTCATTCAAGAAGGAGCAC	
ATG8-pyf3-R	TTACAAGAAGTAGGCACCGGTACCGGGCCCCTATTGACCGAAGGTGTTTTCGC	
pYF3-HA-F	AAGCTTATCGATAGGCCTCCGCGGACTAGTGCCACCATGGTGTACCCA	Construction of an *HA-ATG8* overexpression vector
HA-ATG8-R	TGAGCTCATGCTAGCAACCTCAAGGGATCCAGCGTAATCTGGAACGTCAT	
HA-ATG8-F	AGGTTGCTAGCATGAGCTCATTCAAGAAGGAGC	
ATG8-pYF3-R	TTACAAGAAGTAGGCACCGGTACCGGGCCCCTATTGACCGAAGGTGTTTTCGC	
PYF3-PHB2-F	CAAGCTTATCGATAGGCCTCCGCGGACTAGTGCCGCCATGGAGAACATGAAGAACATGAAGAT	Construction of a *PHB2-Myc* overexpression vector
PHB2-GS-R	GCCACCGCCGCTGCCTCCTCCACCGGATCCTTTCTTCTTGCCGTACGACTGC	
GS-Myc-F	GGATCCGGTGGAGGAGGCAGCGGCGGTGGCGGTAGCGAACAAAAACTAATATCAGAGGAAGATTTG	

Myc-pYF3-R	TTACAAGAAGTAGGCACCGGTACCGGGCCCTTACAGATCTTCTTCTGAAATCAACTTCTG	
pYF3-KoCytc-F	AAGCTTATCGATAGGCCTCCGCGGACTAGTgCCACCATGGTTGACGTTGCCGG	Construction of a *PsCytc-Flag* overexpression vector
Cytc-GS-R	CCACCGCCGCTGCCTCCTCCACCTGGATCCCTCGTTGGTGGCCTCCAT	
GS-Flag-F	GATCCAGGTGGAGGAGGCAGCGGCGGTGGCGGGGGCCCGACTACAAAGACCATGACGGTG	
Flag-pYF3-R	GAAGTAGGCACCGGTACCGGGCCCTTACTTGTCATCGTCATCCTTGTAATCG	
pYF3-AIFB-F	TTATCGATAGGCCTCCGCGGACTAGTGCCACCATGCCTCGCATCCTCATC	Construction of an *AIFB-His* overexpression vector
AIFB-His-R	TTACAAGAAGTAGGCACCGGTACCGGGCCCTCAGTGGTGGTGGTGGTGGTGCTCGAGGTTGGGGACGACGGCG	
pYF3-MC-F	TTATCGATAGGCCTCCGCGGACTAGTGCCACCATGAGCGCCTTCTTCCCC	Construction of a *MC-His* overexpression vector
MC-His-R	TTACAAGAAGTAGGCACCGGTACCGGGCCCTCAGTGGTGGTGGTGGTGGTGCTCGAGGTTCTTCTTGGGGGCGG	

Supplementary Table 11. Sequences of fluorescent and Co-IP tags.

Tag name	DNA sequence	Protein sequence	Category
mCherry	ATGGTGAGCAAGGGCGAGGAGGATAACATGGCCATCATCAAGGAGTTCATGCGCTTCAAGGTGCACATGGAGGGCTCCGTGAACGGCCACGAGTTCGAGATCGAGGGCGAGGGCGAGGGCCGCCCCTACGAGGGCACCCAGACCGCCAAGCTGAAGGTGACCAAGGGTGGCCCCCTGCCCTTCGCCTGGGACATCCTGTCCCCTCAGTTCATGTACGGCTCCAAGGCCTACGTGAAGCACCCCGCCGACATCCCCGACTACTTGAAGCTGTCCTTCCCCGAGGGCTTCAAGTGGGAGCGCGTGATGAACTTCGAGGACGGCGGCGTGGTGACCGTGACCCAGGACTCCTCCCTGCAGGACGGCGAGTTCATCTACAAGGTGAAGCTGCGCGGCACCAACTTCCCCTCCGACGGCCCCGTAATGCAGAAGAAGACCATGGGCTGGGAGGCCTCCTCCGAGCGGATGTACCCCGAGGACGGCGCCCTGAAGGGCGAGATCAAGCAGAGGCTGAAGCTGAAGGACGGCGGCCACTACGACGCTGAGGTCAAGACCACCTACAAGGCCAAGAAGCCCGTGCAGCTGCCCGGCGCCTACAACGTCAACATCAAGTTGGACATCACCTCCCACAACGAGGACTACACCATCGTGGAACAGTACGAACGCGCCGAGGGCCGCCACTCCACCGGCGGCATGGACGAGCTGTACAAG	MVSKGEEDNMAIIKEFMRFKVHMEGSVNGHEFEIEGEGEGRPYEGTQTAKLKVTKGGPLPFAWDILSPQFMYGSKAYVKHPADIPDYLKLSFPEGFKWERVMNFEDGGVVTVTQDSSLQDGEFIYKVKLRGTNFPSDGPVMQKKTMGWEASSERMYPEDGALKGEIKQRLKLKDGGHYDAEVKTTYKAKKPVQLPGAYNVNIKLDITSHNEDYTIVEQYERAEGRHSTGGMDELYK	Fluorescent label
GFP	ATGGGCAAGGGCGAGGAACTGTTCACTGGCGTGGTCCCAATCCTGGTGGAACTGGATGGTGATGTGAACGGGCACAAGTTCTCCGTCAGCGGAGAGGGTGAAGGTGATGCCACCTACGGAAAGCTCACCCTGAAGTTCATCTGCACTACCGGAAAGCTCCCTGTTCCGTGGCCAACCCTCGTCACCACTTTCACCTACGGTGTTCAGTGCTTCTCCCGGTACC	MGKGEELFTGVVPILVELDGDVNGHKFSVSGEGEGDATYGKLTLKFICTTGKLPVPWPTLVTTFTYGVQCFSRYPDHMKQHDFFKSAMPEGYVQERTIF	

	CAGATCACATGAAGCAGCATGACTTCTTCAAGAGCGCC<br>ATGCCCGAAGGCTACGTGCAAGAAAGGACTATCTTCTT<br>CAAGGATGACGGGAACTACAAGACACGTGCCGAAGTC<br>AAGTTCGAAGGTGATACCCTGGTGAACCGCATCGAGCT<br>GAAAGGCATCGATTTCAAGGAAGATGGAAACATCCTCG<br>GACACAAGCTGGAGTACAACTACAACTCCCACAACGTA<br>TACATCATGGCCGACAAGCAGAAGAACGGCATCAAGGT<br>GAACTTCAAGATCAGGCACAACATCGAAGATGGAAGCG<br>TGCAACTGGCGGACCACTACCAGCAGAACACGCCCAT<br>CGGCGATGGCCCTGTCCTGCTGCCGGACAACCATTAC<br>CTGTCCACGCAATCTGCCCTCTCCAAGGACCCCAACGA<br>GAAGAGGGACCACATGGTCCTGCTGGAGTTCGTGACG<br>GCTGCTGGGATCACGCATGGCATGGATGAACTCTACAA<br>GTGTACAGAATCCGTTAACTCCGGAACGCGT	FKDDGNYKTRAEVKFEG<br>DTLVNRIELKGIDFKEDG<br>NILGHKLEYNYNSHNVYI<br>MADKQKNGIKVNFKIRH<br>NIEDGSVQLADHYQQNT<br>PIGDGPVLLPDNHYLST<br>QSALSKDPNEKRDHMVL<br>LEFVTAAGITHGMDELY<br>KCTESVNSGTR	
BFP	ATGAGCGAGCTGATTAAGGAGAACATGCACATGAA<br>GCTGTACATGGAGGGCACCGTGGACAACCATCACTTCA<br>AGTGCACATCCGAGGGCGAAGGCAAGCCCTACGAGGG<br>CACCCAGACCATGAGAATCAAGGTGGTCGAGGGCGGC<br>CCTCTCCCCTTCGCCTTCGACATCCTGGCTACTAGCTT<br>CCTCTACGGCAGCAAGACCTTCATCAACCACACCCAGG<br>GCATCCCCGACTTCTTCAAGCAGTCCTTCCCTGAGGGC<br>TTCACATGGGAGAGAGTCACCACATACGAAGACGGGG<br>GCGTGCTGACCGCTACCCAGGACACCAGCCTCCAGGA<br>CGGCTGCCTCATCTACAACGTCAAGATCAGAGGGGTGA<br>ACTTCACATCCAACGGCCCTGTGATGCAGAAGAAAACA<br>CTCGGCTGGGAGGCCTTCACCGAGACGCTGTACCCCG<br>CTGACGGCGGCCTGGAAGGCAGAAACGACATGGCCCT<br>GAAGCTCGTGGGCGGGAGCCATCTGATCGCAAACATC<br>AAGACCACATATAGATCCAAGAAACCCGCTAAGAACCT<br>CAAGATGCCTGGCGTCTACTATGTGGACTACAGACTGG	MSELIKENMHMKL<br>YMEGTVDNHHFKCTSE<br>GEGKPYEGTQTMRIKVV<br>EGGPLPFAFDILATSFLY<br>GSKTFINHTQGIPDFFKQ<br>SFPEGFTWERVTTYEDG<br>GVLTATQDTSLQDGCLI<br>YNVKIRGVNFTSNGPVM<br>QKKTLGWEAFTETLYPA<br>DGGLEGRNDMALKLVG<br>GSHLIANIKTTYRSKKPA<br>KNLKMPGVYYVDYRLER<br>IKEANNETYVEQHEVAV<br>ARYAGLGGGLN	

	AAAGAATCAAGGAGGCCAACAACGAGACCTACGTCGAG CAGCACGAGGTGGCAGTGGCCAGATACGCGGGCCTGG GCGGCGGCCTGAAC		
Flag	GACTACAAAGACGATGACGACAAAGACTACAAAGA CGATGACGACAAAGACTACAAAGACGATGACGACAAA	DYKDDDDKDYKDD DDKDYKDDDDK	Co-IP label
Myc	GAACAAAAACTAATATCAGAGGAAGATTTGGAACA GAAACTAATCAGTGAAGAAGATTTAGAGCAAAAGTTAAT TTCCGAGGAGGACTTAGAGCAGAAGTTGATTTCAGAAG AAGATCTG	EQKLISEEDLEQKLI SEEDLEQKLISEEDLEQK LISEEDL	
HA	TACCCATACGATGTTCCTGACTATGCGGGCTATCC CTATGACGTCCCGGACTATGCAGGcTCCTATCCATATGA CGTTCCAGATTACGCT	YPYDVPDYAGYPY DVPDYAGSYPYDVPDYA	
MBP	ATGAAAATCGAAGAAGGTAAACTGGTAATCTGGAT TAACGGCGATAAAGGCTATAACGGTCTCGCTGAAGTCG GTAAGAAATTCGAGAAAGATACCGGAATTAAAGTCACC GTTGAGCATCCGGATAAACTGGAAGAGAAATTCCCACA GGTTGCGGCAACTGGCGATGGCCCTGACATTATCTTCT GGGCACACGACCGCTTTGGTGGCTACGCTCAATCTGG CCTGTTGGCTGAAATCACCCCGGACAAAGCGTTCCAGG ACAAGCTGTATCCGTTTACCTGGGATGCCGTACGTTAC AACGGCAAGCTGATTGCTTACCCGATCGCTGTTGAAGC GTTATCGCTGATTTATAACAAAGATCTGCTGCCGAACCC GCCAAAAACCTGGGAAGAGATCCCGGCGCTGGATAAA GAACTGAAAGCGAAAGGTAAGAGCGCGCTGATGTTCAA CCTGCAAGAACCGTACTTCACCTGGCCGCTGATTGCTG CTGACGGGGGTTATGCGTTCAAGTATGAAAACGGCAAG	MKIEEGKLVIWING DKGYNGLAEVGKKFEKD TGIKVTVEHPDKLEEKFP QVAATGDGPDIIFWAHD RFGGYAQSGLLAEITPD KAFQDKLYPFTWDAVRY NGKLIAYPIAVEALSLIYN KDLLPNPPKTWEEIPALD KELKAKGKSALMFNLQE PYFTWPLIAADGGYAFK YENGKYDIKDVGVDNAG AKAGLTFLVDLIKNKHMN ADTDYSIAEAAFNKGET AMTINGPWAWSNIDTSK	Pull down label

	TACGACATTAAAGACGTGGGCGTGGATAACGCTGGCGC<br>GAAAGCGGGTCTGACCTTCCTGGTTGACCTGATTAAAA<br>ACAAACACATGAATGCAGACACCGATTACTCCATCGCA<br>GAAGCTGCCTTTAATAAAGGCGAAACAGCGATGACCAT<br>CAACGGCCCGTGGGCATGGTCCAACATCGACACCAGC<br>AAAGTGAATTATGGTGTAACGGTACTGCCGACCTTCAA<br>GGGTCAACCATCCAAACCGTTCGTTGGCGTGCTGAGCG<br>CAGGTATTAACGCCGCCAGTCCGAACAAAGAGCTGGCA<br>AAAGAGTTCCTCGAAAACTATCTGCTGACTGATGAAGGT<br>CTGGAAGCGGTTAATAAAGACAAACCGCTGGGTGCCGT<br>AGCGCTGAAGTCTTACGAGGAAGAGTTGGCGAAAGATC<br>CACGTATTGCCGCCACTATGGAAAACGCCCAGAAAGGT<br>GAAATCATGCCGAACATCCCGCAGATGTCCGCTTTCTG<br>GTATGCCGTGCGTACTGCGGTGATCAACGCCGCCAGC<br>GGTCGTCAGACTGTCGATGAAGCCCTGAAAGACGCGC<br>AGACT	VNYGVTVLPTFKGQPSK<br>PFVGVLSAGINAASPNK<br>ELAKEFLENYLLTDEGLE<br>AVNKDKPLGAVALKSYE<br>EELAKDPRIAATMENAQ<br>KGEIMPNIPQMSAFWYA<br>VRTAVINAASGRQTVDE<br>ALKDAQT	

Supplementary Table 12. Primers used to generate AIM mutants and domain deletion constructs

**Primer Name**	**Primer Sequences (5' to 3')**	**Description**
PsAF5-AIM1F	GTCCAGCGAGGCTGGCGCGGCTGACCG CCTCATCTCGG	Amplification of the *PsAF5* gene with AIM1 mutation.
PsAF5-AIM1R	AGCCGCGCCAGCCTCGCTGGACTCGGC C	
PsAF5-AIM2F	CTCTGCGCGGATGACGCCCGCGAGAAC GGCGC	Amplification of the *PsAF5* gene with AIM2 mutation.
PsAF5-AIM2R	CTCGCGGGCGTCATCCGCGCAGAGAAT GCACACGCG	
PsAF5-AIM3F	CGCGCGGCTACAGATGCCGATTCGACG GAGAAGTACCAGG	Amplification of the *PsAF5* gene with AIM3 mutation.

PsAF5-AIM3R	CGAATCGGCATCTGTAGCCGCGCGAGGACGTC	
PsAF5-AIM4F	GACGTGGCGATTAATGCCGCGCGTGCGCTTCAC	Amplification of the *PsAF5* gene with AIM4 mutation.
PsAF5-AIM4R	ACGCGCGGCATTAATCGCCACGTCCACGTCCTTGC	
PsAF5-AF	CGAGTGGGGCGCCGAGTGCCTCTTCG	Amplification of the *PsAF5* gene with ANK domain deletion.
PsAF5-AR	GGCACTCGGCGCCCCACTCGCTGGAC	
PsAF5-FF	GCTGCCCCCGGATGACCTGCGCGAGAAC	Amplification of the *PsAF5* gene with FYVE domain deletion.
PsAF5-FR	GCAGGTCATCCGGGGGCAGCGGC	
PsAF5-TF	GCAGGGACGAATGGACCAGGAGGACGAC	Amplification of the *PsAF5* gene with TPR domain deletion.
PsAF5-TR	CCTGGTCCATTCGTCCCTGCACCTCC	

Supplementary Table 13. Primers used for qRT-PCR assays

**Primer Name**	**Primer Sequences (5' to 3')**	**Amplification efficiency**	**R^2 value**
PsAF1-qF	CCGCAGCACCGACCTAATG	103.2%	0.992
PsAF1-qR	GGTTGCCCGTCTTCACAGC		
PsAF2-qF	CGCTCCTGTATGGTGTGCTC	92.9%	0.994
PsAF2-qR	TGCCGCCCTTTGCTTGTAG		
PsAF3-qF	CGACAAGGTGGACAAGGC	91.4%	0.994
PsAF3-qR	CCGCTCGCTGTCAGGTAC		
PsAF4-qF	CCAACCGCAGGGCAACTAC	92.0%	0.999

PsAF4-qR	CGTCCGCTGTCCCGATGG		
PsAF5-qF	TCATTGATATTAACCGCCTCTCC	97.4%	0.998
PsAF5-qR	CGTTCACCTCCCTGTCCTC		
PsAF6-qF	TCTGAAGTTGATTCCTGTTGCTC	92.0%	0.997
PsAF6-qR	ACCCACTCTCCCTGTATTTGTC		
PsAF7-qF	CACACCCGCTCGCTGAAG	91.1%	0.995
PsAF7-qR	GTCGTTGTTGCCCGTAGTG		
PsAF8-qF	AAGACGGTGCCCATTCCTC	93.2%	0.995
PsAF8-qR	TAAGCAGAATCATCAAAGTTAGCG		
PsAF9-qF	CTGGACCCTCTGAAGGACTG	91.0%	0.998
PsAF9-qR	CAGCAAACAGCACAGAATCG		
PsAF10-qF	GCGACTTGCCCGTCATTTC	91.6%	0.981
PsAF10-qR	GCTGCGTCATAGGTAGGTAGG		
PsActin-qF	ACTGCACCTTCCAGACCATC	92.0%	0.998
PsActin-qR	CCACCACCTTGATCTTCATG		
qGmCYP2-F	CCCCTCCACTACAAAGGCTCG	96.8%	0.994
qGmCYP2-R	CGGGACCAGTGTGCTTCTTCA		

137 **Supplementary Table 14. Primers used for mitochondrial quantification by DNA qPCR**

Primer Name	Primer Sequences (5' to 3’)	Description
ATPF01-DL1F	GAACTGGATCTATTGTTAGTGTACCTGTAG	Design of primers for mitochondrial encoded gene *ATPF01*.
ATPF01-DL1R	ACCCTACTTTCTAATTTACTGTTAATTTGACC	

Cytb-DL2F	CTATTTCAGTATAAGGGTATTCAACAGGAC	Design of primers for mitochondrial encoded gene *Cytb.*
Cytb-DL2R	TGGAGGAGTTATTGCTATGTTTGGTTC	

Supplementary Table 15. Accession numbers and annotations of genes utilized in this study.

**Gene name**	**Accession number**	**Domain**
PsAF1	XM_009516948	ANK, FYVE
PsAF2	XM_009527741	ANK, FYVE
PsAF3	XM_009525672	ANK, FYVE
PsAF4	XM_009524157	ANK, FYVE
PsAF5	XM_009527924	ANK, FYVE, TPR
PsAF6	XM_009527856	ANK, FYVE, TPR
PsAF7	XM_009515714	ANK, FYVE
PsAF8	XM_009525821	ANK, FYVE
PsAF9	XM_009541547	ANK, FYVE
PsAF10	XM_009541762	ANK, FYVE
PiAF1	XP_002906256	ANK, FYVE, GPN3
PiAF2	XP_002908070	ANK, FYVE, TPRV
PiAF4	XP_002902184	ANK, FYVE
PiAF5	XP_002907955	ANK, FYVE, TPR
PiAF6	XP_002908012	ANK, FYVE, TPR
PiAF7	XP_002909402	ANK, FYVE
PiAF8	XP_002908612	ANK, FYVE
PiAF9	XP_002905373	ANK, FYVE

PiAF10	XP_002904021	ANK, FYVE
PiAF11	XP_002908680	ANK, FYVE, Myosin motor
PiAF12	XP_002906559	ANK, FYVE, BTB
PiAF13	XP_002908531	ANK, FYVE
PeAF1	RMX_64348	ANK, FYVE, GPN3
PeAF2	RMX_64240	ANK, FYVE, TPRV
PeAF3	RQM_15905	ANK, FYVE
PeAF4	RQM_17842	ANK, FYVE
PeAF5	RQM_16429	ANK, FYVE, TPR
PeAF7	RQM_17962	ANK, FYVE
PeAF8	RMX_69916	ANK, FYVE
PeAF10	RMX_65232	ANK, FYVE, PDZ
PeAF11	RMX_63302	ANK, FYVE, Myosin motor
PeAF12	RMX_66783	ANK, FYVE, BTB
PoAF1	TMW_60743	ANK, FYVE
PoAF2	TMW_63087	ANK, FYVE, TPRV
PoAF3	TMW_57101	ANK, FYVE
PoAF5	TMW_62943	ANK, FYVE, TPR
PoAF7	TMW_60141	ANK, FYVE
PoAF9	TMW_57996	ANK, FYVE
PoAF10	TMW_62273	ANK, FYVE, PDZ
PoAF11	TMW_63803	ANK, FYVE, Myosin motor
PoAF12	TMW_66102	ANK, FYVE, Myosin motor

PoAF13	TMW_55094	ANK, FYVE, BTB
PoAF14	TMW_57092	ANK, FYVE
NiAF1	KAG_7341977	ANK, FYVE, TPR, R-SNARE
NiAF2	KAG_7360962	ANK, FYVE, Myosin motor
NiAF3	KAG_7366568	ANK, FYVE
NiAF4	KAG_7352489	ANK, FYVE
EsAF1	CBJ_32496	ANK, FYVE, Zinc finger
EsAF2	CBJ_48497	ANK, FYVE
GmAF1	KRH_19419	ANK, FYVE
PpAF1	XP_024402157	ANK, FYVE
NtAF1	XP_009620822	ANK, FYVE
AbAF1	XP_006460214	ANK, FYVE, mRNG
RmAF1	CEG_72954	ANK, FYVE, mRNG
UmUpa1	XM_011390610	ANK, FYVE, mRNG
HsANKFY1	NM_016376.5	ANK, FYVE, BTB
DsANKFY1	XP_037728097.1	ANK, FYVE, BTB
McANKFY1	XP_021031236.1	ANK, FYVE, BTB
ScFAB1	NM_001179984.2	Fab1_TCP, FYVE
CeYOTB	NM_066782.8	PH, FYVE
ScVAC1	NM_001180631.3	FYVE, Rab binding domain
HsEEA1	XM_032493636.1	FYVE, Chromosome segregation ATPase
ScVPS27	NM_001183183.1	FYVE, VHS, GAT
PsATG8	XM_009517215	ubiquitin-like (Ubl) domain

PsRab5	XM_009528140.1	Rab5_related
PsRab7	XM_009516921.1	Rab GTPase family 7
PsPHB2	XM_009532734.1	Prohibitin family
PsACTB	XM_009540271	NBD-HSP70/actin family
PsAIF B	XM_009540428	FAD/NAD(P)-binding oxidoreductase
PsMC	XM_009541230	Mito_carrier super family
PsCytc	XM_009531918	Cytochrom_C super family
PsCoadh	XM_009520615	Glutaryl-CoA dehydrogenase
PsATG7	XP_009532228.1	E1_like_apg7
GmCYP2	NP_001344008.1	cyclophilin_ABH_like
