# Supplementary material for: PsAF5 functions as an essential adapter for PsPHB2-mediated mitophagy under ROS stress in Phytophthora sojae
Source: Nat Commun. 2024 Mar 4;15:1967. doi: 10.1038/s41467-024-46290-z (PMC10912746; doi:10.1038/s41467-024-46290-z)
Supplement: Supplementary file 3 — Reporting Summary [file 41467_2024_46290_MOESM3_ESM.pdf]

nature portfolio

Corresponding author(s): Jianqiang Miao, Xili Liu

Last updated by author(s): Feb 1, 2024

Reporting Summary

Nature Portfolio wishes to improve the reproducibility of the work that we publish. This form provides structure for consistency and transparency in reporting. For further information on Nature Portfolio policies, see our Editorial Policies and the Editorial Policy Checklist.

Statistics

For all statistical analyses, confirm that the following items are present in the figure legend, table legend, main text, or Methods section.

n/a	Confirmed	
☐	☒	The exact sample size (*n*) for each experimental group/condition, given as a discrete number and unit of measurement
☐	☒	A statement on whether measurements were taken from distinct samples or whether the same sample was measured repeatedly
☐	☒	The statistical test(s) used AND whether they are one- or two-sided<br>*Only common tests should be described solely by name; describe more complex techniques in the Methods section.*
☒	☐	A description of all covariates tested
☐	☒	A description of any assumptions or corrections, such as tests of normality and adjustment for multiple comparisons
☐	☒	A full description of the statistical parameters including central tendency (e.g. means) or other basic estimates (e.g. regression coefficient) AND variation (e.g. standard deviation) or associated estimates of uncertainty (e.g. confidence intervals)
☐	☒	For null hypothesis testing, the test statistic (e.g. *F*, *t*, *r*) with confidence intervals, effect sizes, degrees of freedom and *P* value noted<br>*Give P values as exact values whenever suitable.*
☒	☐	For Bayesian analysis, information on the choice of priors and Markov chain Monte Carlo settings
☒	☐	For hierarchical and complex designs, identification of the appropriate level for tests and full reporting of outcomes
☒	☐	Estimates of effect sizes (e.g. Cohen's *d*, Pearson's *r*), indicating how they were calculated

Our web collection on statistics for biologists contains articles on many of the points above.

Software and code

Policy information about availability of computer code

Data collection	Bio-Rad CFX manager 4.0 for qPCR data collection and analysis. ChemiScope capture for Western blot images collection. Zen blue 3.4 for fluorescence photos acquisition and analysis.
Data analysis	Clinx software 2.6.1 for Band densities analysis. GraphPad Prism 9 for data analysis and visualization. Multiple sequence alignments were generated with the Jalview 2.11.2.0 program. Phylogenetic trees were constructed with the Maximum likelihood method using MEGA X and further edited using the ITOL v6 tool (https://itol.embl.de). Heat maps were constructed by bioinformatics web site (www.bioinformatics.com.cn) with the qRCP data.

For manuscripts utilizing custom algorithms or software that are central to the research but not yet described in published literature, software must be made available to editors and reviewers. We strongly encourage code deposition in a community repository (e.g. GitHub). See the Nature Portfolio guidelines for submitting code & software for further information.

Data

Policy information about availability of data

All manuscripts must include a data availability statement. This statement should provide the following information, where applicable:

- Accession codes, unique identifiers, or web links for publicly available datasets
- A description of any restrictions on data availability
- For clinical datasets or third party data, please ensure that the statement adheres to our policy

The information about gene involved in this article can be found in Supplementary Table 15. The mass spectrometry proteomics data generated in this study have been deposited in the ProteomeXchange with the identifier PXD044242 [http://proteomecentral.proteomexchange.org/cgi/GetDataset?ID= PXD044242].
The authors declare that all data supporting the findings of this study are available within the article and its Supplementary Information files. Source data are provided with this paper.

Research involving human participants, their data, or biological material

Policy information about studies with human participants or human data. See also policy information about sex, gender (identity/presentation), and sexual orientation and race, ethnicity and racism.

Reporting on sex and gender	N/A
Reporting on race, ethnicity, or other socially relevant groupings	N/A
Population characteristics	N/A
Recruitment	N/A
Ethics oversight	N/A

Note that full information on the approval of the study protocol must also be provided in the manuscript.

Field-specific reporting

Please select the one below that is the best fit for your research. If you are not sure, read the appropriate sections before making your selection.

☒ Life sciences ☐ Behavioural & social sciences ☐ Ecological, evolutionary & environmental sciences

For a reference copy of the document with all sections, see nature.com/documents/nr-reporting-summary-flat.pdf

Life sciences study design

All studies must disclose on these points even when the disclosure is negative.

Sample size	Sample size was determined based on previous publications on similar experimental trials. At least three biological replicates were performed in all experiments.
Data exclusions	No data were excluded from the analyses replication.
Replication	All attempts at replication were successful. The number of replicates is indicated in the corresponding figure legend and/or in the corresponding material and method section.
Randomization	Randomization was used for all the biological experiments.
Blinding	No blinding was done as none of the experiments described in this study involve group allocation during data collection or analyses.

Reporting for specific materials, systems and methods

We require information from authors about some types of materials, experimental systems and methods used in many studies. Here, indicate whether each material, system or method listed is relevant to your study. If you are not sure if a list item applies to your research, read the appropriate section before selecting a response.

Materials & experimental systems

n/a	Involved in the study	
☐	☒	Antibodies
☒	☐	Eukaryotic cell lines
☒	☐	Palaeontology and archaeology
☒	☐	Animals and other organisms
☒	☐	Clinical data
☒	☐	Dual use research of concern
☐	☒	Plants

Methods

n/a	Involved in the study	
☒	☐	ChIP-seq
☒	☐	Flow cytometry
☒	☐	MRI-based neuroimaging

Antibodies

Antibodies used

Anti-Flag antibody (1:8000; AB0008; Abways), anti-GFP antibody (1:5000; AB0005; Abways), anti-MBP antibody (1:5000; AB0029; Abways), anti-ACTB antibody (1:8000; AB2001; Abways), anti-Myc antibody (1:5000; PTM-5390; PTM BIO), anti-mCherry antibody (1:5000; PTM-5619; PTM BIO), anti-ATG8 antibody (1:5000; ab77003; Abcam), anti-ubiquitin (1:2000; AB40290; Abbkine)

Validation

All the label-tag antibodies used in this study were claimed on the manufacturer's website that they could detect all the labeled proteins expressed by eukaryotes normally, and the size of the fusion protein detected in this study was also consistent with the prediction. These label antibodies include anti-Flag, anti-GFP, anti-MBP, anti-Myc, anti-ubiquitin, and anti-mCherry antibodies. Anti-ATG8 antibody can detect purified PsATG8 after prokaryotic expression and detected band size is consistent with predictions (Fig 6b). The sequence similarity of ACTB was more than 90% between Phytophthora sojae with Homo sapiens and Nicotiana tabacum, and size of the protein obtained by westernblot was consistent with the prediction.

Dual use research of concern

Policy information about dual use research of concern

Hazards

Could the accidental, deliberate or reckless misuse of agents or technologies generated in the work, or the application of information presented in the manuscript, pose a threat to:

No	Yes	
☒	☐	Public health
☒	☐	National security
☒	☐	Crops and/or livestock
☒	☐	Ecosystems
☒	☐	Any other significant area

Experiments of concern

Does the work involve any of these experiments of concern:

No	Yes	
☒	☐	Demonstrate how to render a vaccine ineffective
☒	☐	Confer resistance to therapeutically useful antibiotics or antiviral agents
☒	☐	Enhance the virulence of a pathogen or render a nonpathogen virulent
☒	☐	Increase transmissibility of a pathogen
☒	☐	Alter the host range of a pathogen
☒	☐	Enable evasion of diagnostic/detection modalities
☒	☐	Enable the weaponization of a biological agent or toxin
☒	☐	Any other potentially harmful combination of experiments and agents
